# Supplementary material for: Does darkness increase the risk of certain types of crime? A registered report article
Source: PLoS One. 2025 Jun 25;20(6):e0324134. doi: 10.1371/journal.pone.0324134 (PMC12194184; doi:10.1371/journal.pone.0324134)
Supplement: Attachment — Submitted filename: Response to reviewer comments - SUBMITTED.docx [file pone.0324134.s012.docx]

**Response to reviewer comments on “Does darkness increase the risk of certain types of crime? A Registered Report Article” (PONE-D-24-09508)**

We thank all the reviewers for their comments. Our responses to each of their comments are set out below. Please not that references to line numbers refer to the line numbers in the manuscript version with tracked changes.

| **Comment ID** | **Comment** | **Response** |
| --- | --- | --- |
| Reviewer 1 | | |
| 1.1 | In response to the shortcomings of existing research, this article conducts a more detailed study on whether darkness increases the risk of certain types of crime. This article divides the research time period into Case hour and Control hour, and Case hour is divided into CaseDark and CaseDay while Control hour is divided into ControlDark and ControlDay. Then, the article studies the impact of different time periods on different types of crimes, and conducts main analysis and sensitivity analysis; The authors also studied the different impacts of darkness on crime situations in different regions. Overall, the research in this article is comprehensive and detailed, and the hypothesis of this article has been proven through experiments and analysis. | N/A |
| 1.2 | What are the three hypotheses of Article based on? Please provide an explanation. | Justification for the hypotheses is given within the Introduction, e.g. lines 167-241 for hypothesis 1, lines 244-260 for hypothesis 2, lines 261-271 for hypothesis 3.  Further changes to justify and explain the hypotheses would require significant additions to the text and, as agreed with the editor, we are unable to make such significant and substantive changes, as this section of the paper has already undergone peer review and been accepted as part of the published Registered Report Protocol. |
| 1.3 | Is there a reference basis for the calculation formula of OddsRatio and 95% CI? If so, please list them out; If not, please explain the reason for this calculation. | Reference added in relation to odds ratio and confidence interval. |
| 1.4 | The experimental section did not mention the model selection for regression analysis. If a model is used for regression analysis, please introduce the model and its selection criteria; If not, please provide a detailed introduction to the analysis methods in the experimental section. | No regression modelling is used in the analysis of data. Section 2.4 describes how the data are analysed. |
| 1.5 | There are differences between the results of the main analysis and sensitivity analysis. For areas of inconsistency, should the conclusion be based on the main analysis or sensitivity analysis? | The primary conclusions are based on both analyses – they agree in their confirmation of our 3 hypotheses (see Table 9). Where there are differences in the detail of the results of the two analyses we want to avoid saying one should be prioritized over the other. Differences in findings (e.g. that different crimes are highlighted as being at greater risk of committal after dark) are potentially valid, highlighting differences in darkness as a risk factor depending on the time of day (morning vs evening), as is discussed in lines 938-944. |
| 1.6 | The paper states that the results indicate that lighting may help reduce the risk of crime after dark, but the article studies the impact of day and night on crime and does not experimentally prove the impact of lighting on crime (natural light and artificial lighting are different). | The reviewer is correct that we only look at the impact of darkness vs daylight on risk of crime, and not on lighting. This is a precursor step towards understanding the influence of lighting on crime risk after dark due to increased visibility. If no effect of darkness had been found, it would be unlikely that lighting would influence crime risk (through visibility mechanisms), as artificial light provides a smaller increment in light levels and hence visibility than does daylight.  Text has been amended to make clearer that any potential influence of lighting is only suggested by the current results and further work would be required to prove any relationship (see lines 992-994). |
| Reviewer 2 | | |
| 2.1 | General thoughts: The study addresses an interesting research question, the link between ambient light and crime, and has practical implications for practitioners, e.g., allocation of law enforcement resources, ecological interventions involving lighting. The design exploits sharp discontinuities in light/dark due to daylight saving, a plausible identification strategy. The methodological approach is sound: the research questions were pre-registered, past methodological approaches were followed, and various sensitivity checks were carried out. Importantly, the study makes a substantial contribution to the literature by extending Fotios et al. to a different region and more carefully dealing with aoristic crimes (ie, crimes that occurred at an indefinite time). The paper is organized well and clearly written. | N/A |
| 2.2 | The authors make several references to competing ideas for why ambient light might be associated with crime (eg, community pride, routine changes). Early on in the introduction, it would be useful if these ideas were formally laid out. The idea of community pride falls neatly into a couple well-established criminological theories: broken windows theory (first spelled out by Wilson and Kelling) and social disorganization theory (see Shaw and McKay; specifically, factors related to the physical condition of a neighborhood). Findings showing that improved lighting reduces crime might be better explained by signs of improved order in a neighborhood rather than light. Notably, this study can be interpreted as evidence in favor of ambient light as the key ingredient in those interventions, as the variable of light was carefully isolated here. Likewise, routine activity theory might also provide an alternative causal mechanism for the impact of light on crime. This theory is alluded to in several places, but again formally describing it early on will be helpful, as well as specifically discussing how the various elements (motivated offenders, suitable targets, capable guardians) might be implicated. | As agreed with the editor, we are unable to make substantive changes to the Introduction as it was accepted and published as part of the Registered Report Protocol. However, we have added discussion of the different mechanisms for lighting to reduce crime, including reference to the theories referred to by the reviewer, in the Discussion section. See lines 765-780. |
| 2.3 | One big gap in the paper is the lack of an explanation for why ambient light had an effect only on certain offense types (in the main analysis: bike theft, burglary, criminal damage, robbery, vehicle offenses). While I appreciate the authors hesitancy to speculate, in this case I believe it would strengthen the paper to suggest reasons as to why these patterns emerged. Similarly, beyond showing that only certain regions were impacted by changes in light, there does not appear to be an explanation for this finding either. Were there any observable differences in the regions where an effect was found that might explain the finding? | Text added to the Discussion (lines 838-849) and an additional reference added. |
| 2.4 | Suggested sensitivity checks: I was not fully convinced that the approach of using the entire year of data was superior to Fotios et al’s approach of only using data from around the change in daylight saving times. Given the difference in effect sizes between the two papers, I’d be interested to see how much of this was explained by the difference in study timeframes. One possible way to get at this would be to also run your analysis restricted to the periods used in Fotios. Another concern I had was the substantial difference you found when looking at afternoon hours vs morning hours (sensitivity test with different case and control hours). In particular, it made me worry that the distribution might be sufficiently variable throughout the day that your analysis could simple be picking up noise. To address this, it would be useful to conduct a placebo test, ie, run the analysis with ‘case’ hours in daylight throughout the year. A null effect here would be reassuring. | Carrying out an analysis using the daylight saving time clock change method would represent a significant departure from the methods originally proposed in the Registered Report Protocol. The clock change method also has limitations in terms of the sample size of crimes that can be included in the analysis. Fotios et al used crime counts from one week either side of the biannual clock change in the USA, providing 4 weeks of data per year. This compares with a minimum of 40 weeks of data for the whole year approach we have used (after twilight periods have been excluded), which we suggest provides more reliable estimates of the effect of darkness on crime risk.  The suggestion to carry out a ‘placebo’ or null test using case hours that do not change in ambient light condition is a good one. We have carried out null tests, using a selection of case hours that are in daylight throughout the year, and report the results as a post hoc analysis in the Discussion (lines 950-973). No effects are found, as anticipated, suggesting our case and control method is sensitive to the effects of darkness as we assume. |
| 2.5 | The reader would benefit from additional subheadings in the introduction section, eg, limitations of past research on p. 6; current focus on p. 10 | Four subheadings added to Introduction section. |
| 2.6 | One of the major findings from research on the impact of lighting on crime is displacement (eg, see Chalfin, Kaplan, Laforest, 2020). In the discussion section, it would be useful to revisit the issue of displacement in light of your findings (pun intended). Does this study tell us anything useful about the strength of those findings? About how to deal with displacement? | The current study is unable to provide direct evidence related to displacement effects as it examined the influence of darkness on crime rather than the influence of street lighting. However, we have added a comment related to displacement effects when speculating about why we found spatial differences in the effect of darkness (see lines 866-867). We have also added text about the need to consider displacement effects when discussing future research that should examine specifically how lighting influences crime after dark (lines 998-1003). |
| 2.7 | It’s clear that the authors have specialized knowledge about light. For other researchers/policymakers, it might be useful for the authors to spell out with technical detail the minimal dose of light that would be expected to be effective, given the findings here from a natural setting. | Identifying appropriate lighting that might deter crime from being committed after dark is ultimately the goal of our line of research. However, the current study did not examine the influence of lighting on crime and did not measure lighting or light levels and we are therefore unable to provide recommendations about the minimal dose of light to be effective in reducing crime. |
| Reviewer 3 | | |
| 3.1 | This peer review is of the manuscript entitled “Does darkness increase the risk of certain types of crime? A Registered Report Article”. The study uses police-recorded crime data from a UK police agency to test three hypotheses relating to the relationship between darkness and various crime types.  I agreed to review this article because the topic has fascinated me for years. I admire the methods used and believe this work to have used sound analytical decisions and be able to contribute to the field of knowledge. I particularly like that the unit of analysis is space and different crime categories, which is a strength of this research. However, I believe this manuscript needs to be significantly revised to enable readers to grasp the true value of the findings. In what follows I outline my substantive comments, before passing over to my minor comments. I would like to see this work published so my comments are in the spirit of striving to be constructive. | N/A |
| 3.2 | My gravest concern in this manuscript is the lack of adherence to scientific conventions. For example, the authors talk about ‘confirming’ their hypotheses. The philosophy of science is clear (see classic work by Popper) that hypotheses cannot be confirmed. They can only be supported, partially so in many cases. Indeed, the job of the scientist is to try to falsify the hypothesis; although many scientists are not particularly scientific in their conduct. My recommendation is that the authors acquaint themselves with the philosophy of Popperian scientific principles and attenuate the language they use accordingly with regards to hypotheses. | Language about ‘confirmation’ of hypotheses changed to ‘support’ for hypotheses. |
| 3.3 | Relatedly, the purpose of sensitivity testing is to judge whether the findings are predominantly the result of analytical decisions made in the research process. Since the research reported in this research does, indeed, find that results change according to what hour is chosen for the control group, this suggests either that a) patterns are artefacts of analytical decisions rather than of true relationships or b) the control hours used in the sensitivity analysis introduce confounding variables into the analysis. Only when sensitivity analyses do not appreciably affect the findings can you be confident that the original findings are not flukes. | The primary aim of this work was to test three hypotheses, related to the influence of darkness on crime. The main and sensitivity analyses largely agreed in terms of their support for these three hypotheses, so in this respect we suggest the findings are robust to the choice of case and control hour.  The detail of the results in the main and sensitivity analysis did vary however, as highlighted by the reviewer. We discuss potential reasons for these differences in the Discussion (see lines 935-949). The differences highlight the potential that the effect of darkness on crime may vary depending on whether the darkness is in the morning or evening.  Following a comment from reviewer 2 (see comment 2.4), we have also carried out a post hoc ‘null’ test analysis, checking the assumption that if the light condition does not change during a case hour (i.e. remains in daylight throughout the year), no effect of darkness would be found. As expected, no effects were found for these null tests, giving some confidence that the case and control method is able to successfully identify effects of darkness. See lines 950-973. |
| 3.4 | Another hallmark of science is that science writing should be precise and concise. This is a developmental skill that is honed over one’s career. I’m reminded of the pithy observation: “If I had more time I would have written you a shorter letter”. The aspiration in scientific writing is to ‘say it right, say it once’, not to repeat. The manuscript I reviewed was severely overwritten, with needless repetition throughout and tangential information (e.g., illuminance under twilight conditions is just one example) included. Omitting twilight conditions in the analysis was probably wise, so to have an extended discussion about twilight is puzzling. Likewise, the discussion about crime recoding processes can be condensed into one sentence. The same applies to data excluded from analysis. In Section 2.7 all you have to say here is that a coding script was written a priori based on knowledge of the data structure and passed to the data owners to run. There are countless other examples where important points can be condensed into one sentence and severely condense the manuscript.  If this work started life as a (laudible) student project then time now should be invested in refining the writing to remove redundancy and to make every word in every sentence be doing the heavy lifting. I believe the current manuscript could be reduced by two-thirds in words and still communicate the same crucial information. You may like to consult a writing book (e.g., my current favourite is John Eck’s ‘Writing with sweet clarity) to help you to edit your work until it is clear and concise. | We agree that science writing should be precise and concise. It should also provide sufficient detail and context to enable the reader to understand the background and what has been done, allowing them to follow the analytical decisions that have been made and to replicate the analysis if necessary. We therefore prefer to keep detail within the manuscript, this being preferable to having a shorter manuscript that potentially lacks critical information required to allow a reader to follow our arguments and justification of analytical decisions, and to reproduce or replicate our findings.  We also note the comment from reviewer 2 (comment 2.1) that “The paper is organized well and clearly written”. We have, however, made a number of small revisions to the manuscript to improve clarity of text. We are unable to substantially revise the text in the Introduction and Method however, as these were accepted and published as part of the Registered Report Protocol. Avoiding significant changes to these previously reviewed and published sections has been agreed with the editor. |
| 3.5 | A further hallmark of science is that inferences are used in reasoning. I am confused as to why ‘fear of crime’ is discussed as thoroughly as it is in the manuscript. Fear of crime is important and the vastness of the literature is not adequately covered, but the relation of fear of crime to crime (the topic of inquiry) is nebulous. For example, the premises of why darkness is likely to inspire fear of crime are not logically linked to the conclusion that darkness does indeed inspire fear of crime. And this is not linked to crime occurrence in any way. Either specify the causal relationship between fear of crime and crime more clearly or de-emphasise the fear of crime narrative. In my view fear of crime is tangential and a scientific paper only focuses on that which is central. | The topic of fear of crime was discussed to provide context to the current study. It is a common belief that darkness can increase the risk of being victimized, and that street lighting can help mitigate this risk. These perceptions relate to the fear of crime. Our study examines whether such fear of crime after dark is justified or not. It is also relevant when discussing the role of lighting to reduce crime – even if it does not reduce crime, it may still reduce fear of crime, which can have societal benefits. In addition, to remove or substantially amend the discussion of fear of crime would represent a significant revision to the Introduction section, which has already been through peer review and accepted for publication as part of the Registered Report Protocol. As agreed with the editor, it is not appropriate for us to make such significant and substantive changes to the material already published in the Protocol. |
| 3.6 | Scientific writing also requires all claims to be substantiated. Examples of where this has not been done are: • Page 12: street lighting consumes energy and impacts on nocturnal environment. • Page 12: it is not clear how land use, economic conditions and temporal factors have been chosen to illustrate the point of confounders to lighting influences. You need to articulate the argument more logically for the reader to be able to follow. | As agreed with the editor, we are unable to make substantive changes to the Introduction as it was accepted and published as part of the Registered Report Protocol. |
| 3.7 | Synthetic data is the conventional term for what you call ‘fictional data’. There are contemporary criminologists writing about this whose work would be worth reading so you understand the implications of providing such data. | Text revised to replace ‘fictional’ with synthetic. We do note however that this revision means a different term is being used than that used in the original Registered Report Protocol. For example, two of the Supporting Information files (S3 and S4) in the published Protocol refer to ‘fictional’ data. We are happy to revert back to using ‘fictional’ data in the current manuscript if the editor would prefer us to keep terms consistent with the original Protocol. |
| 3.8 | Page 10, line 102, reference [13] used precise crime data and hence undermines the trend you present earlier in the manuscript. | If the reviewer is referring to our statement “The crime data used in the study did not include information about the time of occurrence meaning it was not possible to distinguish between crimes that occurred after dark or during daylight.”, this was a criticism of one particular study (Steinbach et al, 2015), we were not suggesting that all studies of crime and light fail to use precise crime data. |
| 3.9 | Given the mention of economic and environmental concerns of lighting earlier in the manuscript, I was surprised that the following sentence did not acknowledge these trade-offs on page 15: “Good lighting could be considered that which offsets any increase in criminal activity after dark.” | We have added the text: “In the context of crime reduction…”, to acknowledge we are referring to any influence lighting has on crime (see line 287). We have also added text to the Conclusion to acknowledge that any benefits of lighting for crime reduction should be balanced against potential negative impacts of lighting (lines 996-998). |
| 3.10 | Page 16, lines 297-302, these justifications should probably be in the methods section. | As agreed with the editor, we are unable to make substantive revisions to the Introduction and Method as they were accepted and published as part of the Registered Report Protocol |
| 3.11 | Table 1: Would potentially make this clearer at first glance if the case and control hours/times were in the table. | Times of case and control hours are provided in Table 2. |
| 3.12 | Table 3: I do not think this table necessary. You could just say crime data included the offence code, the MSOA code/name, the committed from/to date and time—and how any variables were transformed—instead. In one sentence. | As agreed with the editor, we are unable to make substantive changes to the Method as it was accepted and published as part of the Registered Report Protocol |
| 3.13 | Page 24: It is not clear here how Fisher's exact test is being applied. is it for the 2x2 table of case/control light/dark? A little more foregrounding of this test would be useful in the methods section. | Text added to clarify Fisher’s exact test is applied to the 2x2 contingency table of crime counts. |
| 3.14 | Page 18, 31: it is not clear why one would not want confidence intervals to overlap. This needs a logical argument, expressed using statistical theory, to explain why it matters. | Text added to clarify that non-overlapping CIs indicate a significant difference, and reference about CIs also added. |
| 3.15 | It would be useful context to know what proportion of crime fell into each of the case and control hours used in the analysis prior to Table 1. | Text added to section 3.1 |
| 3.16 | Page 26: I did not understand the sentence: The proportion of crimes in the control hour when it was in darkness was 50% of all crimes recorded in the control hour. Maybe rephrase? | Sentence revised for clarity. |
| 3.17 | Cite the protocol document when you first mention it in the manuscript. In addition, consider referring to the protocol document throughout for information that is contained there, so your registered report just documents the new information. Presently, there is a lot of self-plagiarism between the protocol and registered report. | We first mention the Registered Report Protocol at the end of the Introduction, and provide a reference to the Protocol at that point.  We have followed the approach that previous Registered Report Articles published in Plos One have followed, essentially restating the text from the Registered Report Protocol in the subsequent Registered Report Article. We understand that the Protocol would be linked to from the Article page on the Plos One website, if accepted for publication. |
| 3.18 | Figure 2: a vertical line at the 1 marker would help intuitive interpretation of this plot. | Vertical line added to Figures 2 and 4 |
| 3.19 | Page 31: you might benefit from leading with the finding: 25 MSOAs had odds ratios with CIs that did not overlap with one. These all had odds ratios above one and are highlighted in the map of MSOAs in Figure 3. | Text amended |
| 3.20 | Page 32: As a reader I am left wondering what is it about these MSOAs that mean darkness facilitates crime? This is a novel part of the analysis and deserves more attention. What land use/populations/conditions are creating situations there that are encouraging crime? Presently, all you produce is descriptives, not explanations for why darkness might be important at these locations in particular. What do they have in common? | We agree this is an interesting question that warrants further investigation but explaining spatial differences was not the initial goal of this work. We have however added text to speculate about possible explanations for the variation in odds ratios between MSOAs (see lines 859-872) |
| 3.21 | Figure 3: legend, scale bar, north arrow, inset map regarding study area? These are all essential cartographic features of a map. | Figure updated |
| 3.22 | Page 35: is it worth saying that shoplifting is predominantly committed in the daytime (according to shoplifters the early afternoon hours are the perfect level of footfall in shops for optimal conditions for shoplifting) and this may explain your results? | We do not follow how this would explain the result that the odds ratio for shoplifting was significantly below 1.0. The odds ratio assesses the risk of crime occurring after dark relative to daylight. It controls for the time of day, so whether shoplifting is predominantly committed during early afternoon hours should not influence the odds ratio obtained. |
| 3.23 | Page 37: I’m not convinced you can call your treatment of aoristic crime a ‘method’. To my mind it is a set of decision rules for analysis. | Text amended |
| 3.24 | Page 40: I do not share your confidence that excluding crimes that were reported over 1 hour will not upwardly or downwardly bias your results. I imagine this will play out differently over crime types. For example, going on international trends it is likely that the daytime period is when people report burglary, but the nighttime when they report vehicle crime has happened. If this holds in your data, these patterns would bias your results but cancel each other out in aggregate. I would like to see a fuller analytic exploration of this before making a sweeping statement that “we therefore assume does not confound the results in any particular direction. | We agree different crime types will predominantly occur at different times of the day. If an aoristic crime like burglary is more likely to occur during the daytime, the number of such crimes we included in the control hours (daytime hours, between 13:00 and 15:59 in the main analysis) could be an underrepresentation of the true number, if many of these crimes were excluded because the time window was greater than one hour. However, we believe this potential underrepresentation would occur equally in both the controlDay period (when the case hour is in daylight) and the controlDark period (when the case hour is in darkness). Therefore the effect of excluding burglaries that had a time window larger than one hour would not confound the overall result for burglaries, because these exclusions are expected to fall equally across the comparative periods.  Text added to further explain why we do not think confounding of results will occur, see lines 893-899. |
| Reviewer 4 | | |
| 4.1 | The authors present a very comprehensive examination of how daylight influences crime. There is one limitation/framing issue that I would like to see the authors consider ahead of publication. The lighting of the area that the specific crimes took place in was not measured, only the level of daylight was accounted for. This is important as much of the prior literature cited, and the implications discussed, consider street lighting. The current study is therefore not so interested in the risk factor of darkness as it is the protective factor of daylight. This distinction between daylight, light, and darkness should be explored more in the discussion in particular. | We would prefer to continue framing the research in terms of the potential increased risk darkness might bring, relative to daylight, rather than the protective factor of daylight. Although some of the prior literature and associated implications relate to artificial lighting, this is with the premise that darkness may increase the risk of crime and lighting is being assessed to see whether it can offset this increased risk. One goal of our research is to empirically assess the premise that darkness increases risk of crime, hence why we would prefer to continue framing it in relation to darkness and an increase in risk rather than daylight and a reduction in risk. A further goal of this research is to quantify any increase in risk caused by darkness, as this quantification can then be used to assess the efficacy of lighting in reducing risk of crime after dark. Such a quantification of the risk caused by darkness would not be logical or achievable if the research were framed around the protective factor of daylight. We believe assessing the potential increase in risk posed by darkness is a necessary first step before assessing the impact lighting could have on crime. |
